# Efficient and Broadband Emission in Dy^3+^-Doped Glass-Ceramic Fibers for Tunable Yellow Fiber Laser

**DOI:** 10.3390/nano13091558

**Published:** 2023-05-05

**Authors:** Qianyi Chen, Minbo Wu, Puxian Xiong, Yajing Zhao, Shuhang Tian, Yao Xiao, Yongsheng Sun, Dongdan Chen, Shanhui Xu, Zhongmin Yang

**Affiliations:** 1 State Key Laboratory of Luminescent Materials and Devices, Guangdong Engineering Technology Research Center of Special Optical Fiber Materials and Devices, Guangdong Provincial Key Laboratory of Fiber Laser Materials and Applied Techniques, Institute of Optical Communication Materials, South China University of Technology, Guangzhou 510641, China; 202121022299@mail.scut.edu.cn (Q.C.); 201810107807@mail.scut.edu.cn (M.W.); msxiong.puxian@mail.scut.edu.cn (P.X.); 202021021427@mail.scut.edu.cn (Y.Z.); 201921020203@mail.scut.edu.cn (S.T.); 202121022326@mail.scut.edu.cn (Y.X.); 202021021417@mail.scut.edu.cn (Y.S.); flxshy@scut.edu.cn (S.X.); yangzm@scut.edu.cn (Z.Y.); 2 School of Physics and Optoelectronics, South China University of Technology, Guangzhou 510641, China

**Keywords:** glass-ceramic fiber, broadband, yellow emission

## Abstract

Yellow lasers are of great interest in biology, medicine and display technology. However, nonlinear emission of near-infrared lasers at yellow still presents particularly complex optical alignment to date. Here, to the best of our knowledge, we demonstrate the fabrication of a NaLa(WO_4_)_2_: Dy^3+^ glass-ceramic fiber (GCF) for the first time. More importantly, the emission band of the GCF, which is around 575 nm, has a wide full-width half maximum (FWHM) of 18~22 nm, which is remarkably larger than that of the Dy^3+^-doped YAG crystal (<7 nm). The precursor fiber (PF) was drawn using the molten core drawing (MCD) method. In particular, benefiting from the in situ nanocrystals fabricated in the amorphous fiber core after thermal treatment, the resultant glass-ceramic fiber exhibits a five-times enhancement of luminescence intensity around 575 nm, compared with the precursor fiber, while retaining its broadband emission. Overall, this work is anticipated to offer a high potential GCF with prominent bandwidth for the direct access of a tunable yellow laser.

## 1. Introduction

Nowadays, yellow light lasers operating at 565–590 nm have attracted extensive attention in various fields, for example, in the biomedical area, where yellow light lasers can be used for the treatment of vascular diseases due to hemoglobin’s high absorption of yellow light at wavelengths [1,2,3]. However, the output of a yellow laser is relatively difficult, since it is mainly based on high maintenance dye lasers or complex nonlinear frequency transformations such as a sum frequency generation [4], frequency doubling [5], four-wave mixing [6] and Raman conversion [7]. By contrast, direct access of a yellow laser by using a laser diode (LD) to pump rare-earth (RE)-ion doped fibers can leave out the complicated nonlinear optical processes for higher integration and has a high heat dissipation performance. Thereinto, a trivalent rare-earth dysprosium (Dy^3+^) ion is an attractive candidate, whose ^4^F_9/2_ → ^6^H_13/2_ transition can produce strong yellow fluorescence [8]. In 2000, Limpert et al. were the first to report on a Dy^3+^-doped ZBLAN fiber pumped by an argon ion laser, and they were able to resoundingly obtain a CW yellow laser [9]. In recent years, benefiting from the rapid development of high-power blue semiconductor (e.g., GaN, InGaN) laser diodes [10], an improved yellow performance has been realized in Dy^3+^-doped fibers [11]. In 2021, Zou et al. reported that Dy^3+^-doped ZBLAN fibers pumped by GaN laser diodes realized yellow lasers with a maximum output of 1.12 W at 575 nm [12]. Thereafter, ps-level mode-locked yellow fiber lasers have been demonstrated with Dy: ZBLAN using dissipative soliton resonace (DSR) [13]. These reports highlight the favorable advantage of Dy^3+^-doped ZBLAN fibers in compact yellow lasers. However, up to now, Dy^3+^-doped fiber yellow lasers have mainly been limited to fluoride fibers [9,11,12,13,14] which may not be helpful in high power lasers due to their relatively poor chemical stability and low laser-induced damage threshold.

Traditional Dy^3+^-doped laser crystals, such as YAG [15] and LiLuF_4_ [16], have been recognized as the mainstream gain materials for yellow lasers due to their high physical and chemical stability and high mechanical strength. However, the emission bandwidth at a yellow wavelength are usually <7 nm, which limits their applications in tunable or ultrafast lasers. Moreover, the laser crystal preparation is still demanding and expensive. In recent decades, disordered crystals have been widely studied for their laser output properties. By exploiting the significant merits of their non-uniformly broadening spectrum and long phonon mean free path, disordered crystals are regarded as a promising gain medium to effectively generate femtosecond lasers [17] and tunable lasers [18]. What is more, disordered crystals are beneficial to be Dy^3+^ ion-doped host materials since they (a) broaden the absorption spectrum conducive to the improvement of the pump absorption efficiency; and (b) broaden the emission spectrum to realize tunable yellow lasers. Thereinto, disordered crystal composite fibers that combine the advantages of glass fiber and laser crystals may be the ideal choice for tunable yellow fiber lasers. In addition, bimetallic tungstate as a class of promising disordered emitters demonstrates exceptional performance in visible and near-infrared ranges [19,20,21]. Notably, visible lasers have been realized in KRE(WO_4_)_2_ (with RE = Gd or Y) crystals [22] and the stable passive Q-switching of a Yb^3+^: NaY(WO_4_)_2_ laser has been demonstrated [23]. Based on the study of bimetallic tungstate in yellow emission, Dy^3+^-doped NaLa(WO_4_)_2_ with encouraging physical properties has attracted our attention [24]. Noteworthy, due to the composite fiber preparation technology, which is still highly challenging, there are still no reports on Dy^3+^-doped disordered crystal composite fibers.

Herein, a silica glass clad fiber containing a NaLa(WO_4_)_2_:Dy^3+^ disordered nanocrystal core was drawn using the molten core method. Broadband yellow emissions around 575 nm (bandwidth: 18~22 nm) were obtained in the GCFs. The fiber core material of Dy^3+^-doped NaLa(WO_4_)_2_ were synthesized using a high temperature solid-state reaction method and PFs were prepared using the MCD method. What is more, the emission intensity of GCFs was significantly enhanced by up to five times compared to that of as-drawn precursor fibers. These results stressed that the Dy^3+^-doped NaLa(WO_4_)_2_ GCF is a promising gain material for tunable yellow lasers.

## 2. Materials and Methods

### 2.1. Fiber Core Material Synthesis and Fiber Preparation

A series of NaLa_1−*x*_(WO_4_): *x*Dy^3+^ (*x* = 0, 0.04, 0.05, 0.06) powders were prepared using the solid-state reaction method. Commercial raw materials for Na_2_CO_3_ (purity ≥ 99.9%, Macklin, Shanghai, China), La_2_O_3_ (purity ≥ 99.99%, Aladdin, Shanghai, China), WO_3_ (purity ≥ 99.99%, Aladdin, Shanghai, China) and Dy_2_O_3_ (purity ≥ 99.99%, Aladdin, Shanghai, China) were accurately weighed and mixed and were then placed in a muffle furnace and synthesized at 1100 °C for 9 h under an air atmosphere. These samples were cooled down to room temperature and ground to a fine powder for further use. Subsequently, NaLa_1−0.05_(WO_4_): 0.05Dy^3+^ powders were pressed into strip-shaped blanks with the appropriate amount of PVA solution as an adhesive, followed by being put back into the muffle furnace and sintered at 1100 °C for 8 h. The prepared NaLa_1−0.05_(WO_4_): 0.05Dy^3+^ ceramic was ground and polished into a rod with a diameter of 3.8 mm, then was put into a silica glass tube with an outer diameter of 25 mm and inner diameter of 4 mm to assemble the preform. NaLa(WO_4_)_2_: Dy^3+^ ceramic derived fibers were drawn at 1980 °C in a commercial fiber drawing tower. Afterwards, we attempted to tailor the heat-regime during crystallization in the PF cores. We propose that the rational control of the crystallization path helps GCF luminescence enhancement.

### 2.2. Molecular Dynamics Simulations

Molecular Dynamic (MD) simulations were employed using a set of interatomic potentials in the Buckingham form [25,26,27] in order to understand the crystallization process in the GCF cores. A cubic simulation box was constructed to consist of 9240 atoms with a composition of 90NaLa(WO_4_)_2_-10SiO_2_. Initial atomic coordinates were randomly generated by the program PACKMOL (Version 16.0.60) [28]. The simulation protocol was performed using the LAMMPS [29] package and initiated with relative equilibration 0.5 ns at 3000 K to remove the memory effects of the initial structure. Then, the system was gradually cooled down from 3000 to 300 K with a nominal cooling rate of 1 K/ps [30]. At 300 K, the system was equilibrated for 1 ns to relax the structure. Finally, the system equilibrated for 10 ns at 1233 K. During the final 500,000 steps, atomic configurations were recorded at every 1000 steps for further calculations. These processes were run with a step of 2 fs in the canonical (NVT) ensemble.

### 2.3. Characterization Methods

The X-ray diffraction (XRD) patterns of the NaLa_1−*x*_(WO_4_): *x*Dy^3+^ were determined by an X’Pert PRO X-ray diffractometer (PANalytical, Almelo, The Netherlands) using Cu Kα (λ = 1.5418 Å) radiation. The excitation, emission spectra and lifetime measurements of NaLa_1−*x*_(WO_4_): *x*Dy^3+^ were measured by an Edinburgh FLS920 spectrometer (Livingston, UK). The microstructure of the PF cross section was studied using scanning electron microscopy (SEM, JSM-2010, Tokyo, Japan) equipped with an energy dispersive X-ray spectrometer (EDS). The Raman spectra of fibers were characterized by a Micro-Raman spectrometer (Renishaw inVia, Pliezhausen, Germany) under the excitation of a 532 nm laser. The fibers were excited by a 450 nm laser diode (LD) with a maximum power of 12 W. The optical properties of fibers were evaluated on an optical microscope (Nikon, LSH-H100C-1, Tokyo, Japan) equipped with a charge-coupled device (CCD, Nikon, Tokyo, Japan). The fiber optic spectrometer (Ocean optics, Maya2000, Pro, Orlando, FL, USA) was employed to record the emission spectra of the fibers under different pump powers. To identify the morphology of nanocrystals in GCFs, high-resolution transmission electron microscopy (HR-TEM) photography was measured by transmission electronic microscopy (TEM, FEI Talos F200x, Waltham, MA, USA). A cutback measurement was carried out on a 1 m long fiber to determine the propagation loss at 532 nm.

## 3. Results and Discussion

### 3.1. Phase Structure and Photoluminescence Properties

**Figure 1:**
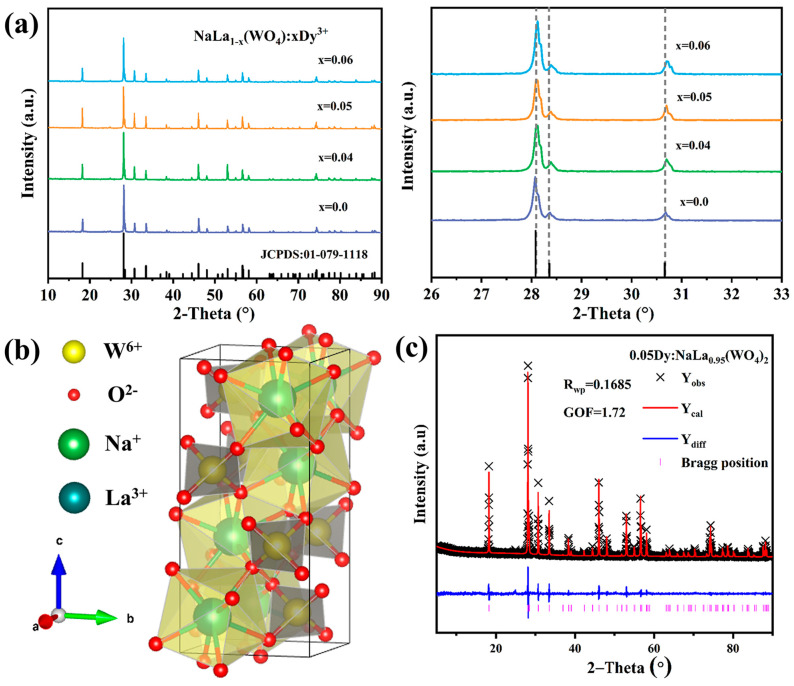
(**a**) XRD patterns of NaLa_1−*x*_(WO_4_)_2_: *x*Dy^3+^ (*x* = 0, 0.04, 0.05, 0.06) and the JCPDS standard card of NaLa(WO_4_)_2_. (**b**) Schematic representation of the NaLa(WO_4_)_2_: Dy^3+^ crystal structure. (**c**) Rietveld refined mapping of NaLa_0.95_(WO_4_)_2_: 0.05Dy^3+^ XRD data.

The X-ray diffraction patterns of the NaLa_1−*x*_(WO_4_): *x*Dy^3+^ (*x* = 0, 0.04, 0.05, 0.06) are presented in Figure 1a. All of the observable diffraction peaks match well with the standard card of NaLa(WO_4_)_2_ (JCPDS No.01-079-1118) and no extra peak or impurity appears in the patterns, which ensures the phase purity. As shown in Figure 1b, the bimetallic tungstate NaLa(WO_4_)_2_ belongs to the tetragonal structure with I_41/a_ space group, and the lattice constants a = b = 5.358 Å, c = 11.656 Å. In this structure, the W^6+^ ions are coordinated with four O^2-^ ions, forming typical [WO_4_]^2−^ tetrahedrons. Meanwhile, the Na^+^ and La^3+^ ions are distributed in a ratio of 1:1 between the [WO_4_]^2−^ layered groups, which results in a disordered structure. Considering the similar ionic radii, Dy^3+^ ions tend to replace the La^3+^ positions in the host. Upon increasing the Dy^3+^ doping concentration, the XRD peaks appear to shift to a larger angle, which can be confirmed by the XRD patterns given in Figure 1a. The Rietveld refinement of the XRD patterns of the NaLa_0.95_(WO_4_)_2_: 0.05Dy^3+^ sample are shown in Figure 1c. The reliability parameters of R*_wp_* = 13.85% and GOF (goodness of fit) = 1.72 were obtained. The individual atomic positions of the final refinement are listed in Table 1 and Table 2, respectively. It is noted that the refined lattice constants a = b = 5.355 Å, c = 11.649 Å correspond to NaLa_0.95_(WO_4_)_2_: 0.05Dy^3+^, which further indicates that Dy^3+^ has been incorporated into the NaLa(WO_4_)_2_.

**Figure 2:**
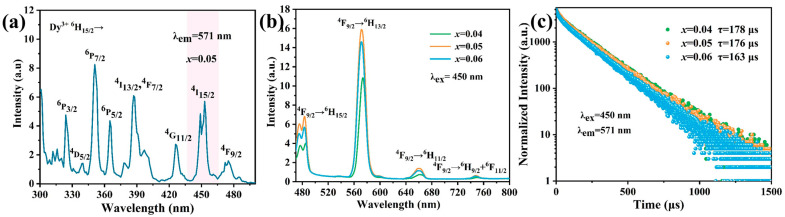
(**a**) Excitation spectra of NaLa_0.95_(WO_4_)_2_:0.05Dy^3+^. (**b**) Emission spectra and (**c**) lifetime decay curves of NaLa_1−*x*_(WO_4_)_2_:*x*Dy^3+^ (*x* = 0.04, 0.05, 0.06).

Figure 2a shows the excitation spectra of the NaLa_0.95_(WO_4_)_2_: 0.05Dy^3+^ sample. In the blue band, there is a strong absorption band which peaked at 450 nm, which is attributed to the ^6^H_15/2_→^6^I_15/2_ transition of Dy^3+^. The emission spectra of the NaLa_1−*x*_(WO_4_)_2_: *x*Dy^3+^ (*x* = 0.04, 0.05, 0.06) samples at room temperature are exhibited in Figure 2b. Four emission peaks at 483, 572, 661 and 751 nm are observed under 450 nm excitation, which are caused by the ^4^F_9/2_→^6^H_15/2_, ^4^F_9/2_→^6^H_13/2_, ^4^F_9/2_→^6^H_11/2_, and ^4^F_9/2_→^6^H_9/2_ + ^6^F_11/2_ transitions of Dy^3+^, respectively. Figure 2c presents the integral emission intensities of NaLa_1−*x*_(WO_4_)_2_:*x*Dy^3+^ corresponding to different Dy^3+^ concentrations. It is noted that the yellow emission intensity of NaLa_1−*x*_(WO_4_)_2_:*x*Dy^3+^ reaches a maximum value at *x* = 0.05. As the concentration of the Dy^3+^ increases further, the intensity of the Dy^3+^ emission decreases due to the concentration quenching effect. Figure 2c shows the lifetime decay curves for NaLa_1−*x*_(WO_4_)_2_:*x*Dy^3+^ (*x* = 0.04, 0.05, 0.06) and their lifetime was calculated by using Equation (1) [31], as follows:

(1) $$I\left(t\right)=I_{o}+A_{1}\exp\left(-t/\tau_{1}\right)+A_{2}\exp\left(-t/\tau_{2}\right)$$

The average life value can be subsequently found as follows (2):

(2) $$\tau^{*}=\frac{A_{1}\tau_{1}^{2}+A_{2}\tau_{2}^{2}}{A_{1}\tau_{1}+A_{2}\tau_{2}}$$

where *I*(*t*) refers to the time-dependent intensity, *τ* represents the lifetime values of different decay components and *A*_1_ and *A*_2_ are fitting constants. It can be seen that the average fluorescence lifetime shorts from 178 μs to 163 μs as the Dy^3+^ concentration increases from 0.04 to 0.06, which is ascribed to the strengthened cross relaxations of Dy^3+^.

### 3.2. Dynamics Analysis of Crystallization Process in Fiber

**Figure 3:**
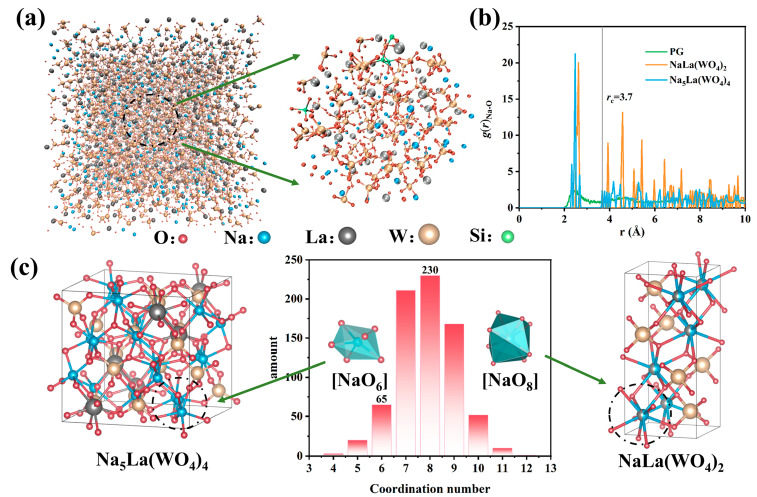
(**a**) Snapshot of MD simulation structure of the fiber core and part of structure enlarged. (**b**) Radial distribution function of Na-O in simulation structure, NaLa(WO_4_)_2_ and Na_5_La(WO_4_)_4_. (**c**) The distribution of coordination number for Na and the crystal structure of Na_5_La(WO_4_)_4_ and NaLa(WO_4_)_2_, respectively.

The multi-material fiber drawing at high temperatures generally processes mutual element diffusion between the cladding and core. Under the fast co-solidification process, the fiber core is formed by a new component of amorphous glass and the nanocrystal growth process is affected by the distribution of the surrounding elements. We chose a commercial silica glass with excellent thermos-mechanical properties as the fiber cladding and systematically studied the crystallization potential of the fiber core region. Figure 3a emulates the bulk structure of the PF core after thermal treatment. A visual inspection of the atomistic configurations reveals that the main body is consistent with a [WO_4_] and [SiO_4_] tetrahedron, while La and Na ions are randomly distributed in the interstices of the tetrahedral. This indicates that the atomic arrangement of the fiber core shows a certain order. We further analyzed the structure in relation to the crystal. By comparing the radial distribution functions (RDF) of Na-O in the above structure and NaLa(WO_4_)_2_/Na_5_La(WO_4_)_4_, it can be observed that the peak positions of both crystals are quite close to the first peak of the glass (Figure 3b). The short-range interactions were evaluated using the cut-off distance of the Na atom as 3.7 Å, and further discuss surrounding ligands of the Na. Notably, these two compounds are intrinsically different in the number of coordination for the Na atoms. According to the statistics in Figure 3c, the coordination number of Na atoms is mainly concentrated between 7–9 and reaches a maximum at 8, which corresponds to the [NaO_8_] octahedral structure in NaLa(WO_4_)_2_. On the contrary, the number of hexahedra corresponding to Na_5_La(WO_4_)_4_ is much lower, illustrating that NaLa(WO_4_)_2_ was preferential to crystallization compared to Na_5_La(WO_4_)_4_. The above analysis guided the design of the components of the precursor fiber and demonstrated the feasibility of the system.

### 3.3. Fiber Characterization and Yellow Emission Performance

**Figure 4:**
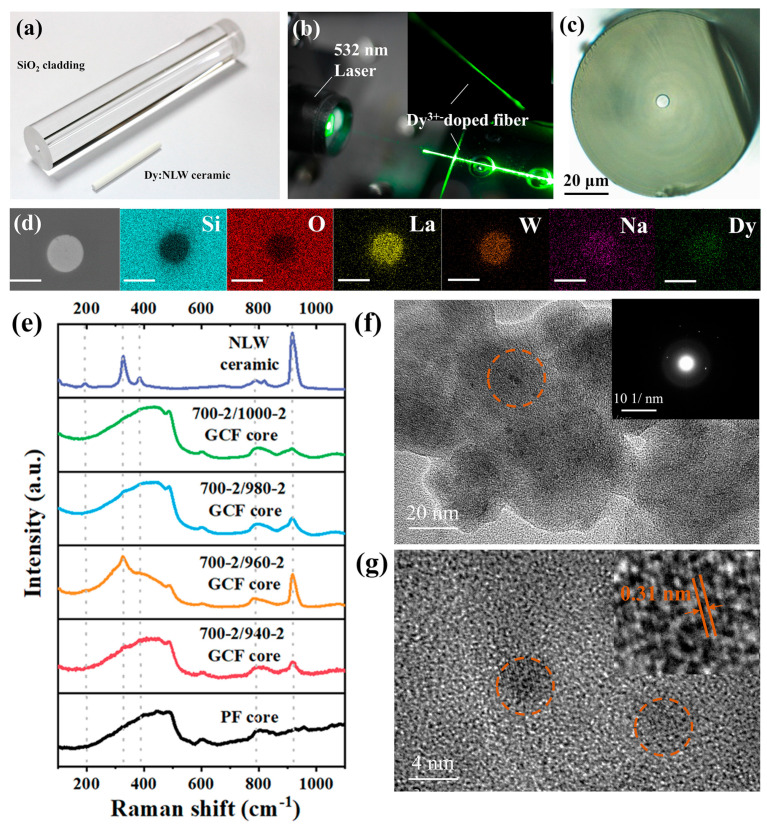
(**a**) Photograph of preform and (**b**) the fiber coupled with green laser beam. (**c**) Cross sectional microscope image of PF and (**d**) its SEM image and EDS mappings of different elements (scale bar: 8 μm). (**e**) Micro-Raman spectra of the PF, GCFs fiber and NaLa(WO_4_)_2_ ceramic. (**f**) Micrograph of TEM and the inset is the (SAED) pattern (**g**) HR-TEM image (inset: lattice structure) of GCFs heat-treated at 700 °C-2 h/960 °C-2 h. The dotted cirle highligh the areas of nanocrystals.

Guided by the above design, as depicted in Figure 4a, a preform with a core-cladding structure achieved by loading a NaLa_0.95_(WO_4_)_2_:0.05Dy^3+^ ceramic rod into a silica glass tube was used. Considering the huge difference between the core melting temperature (~1250 °C) and the softening temperature (~1900 °C), the MCD method was utilized for fiber preparation. Figure 4b presents the constructed fiber coupled with a green laser beam, demonstrating the excellent transparency of the fabricated fiber. Figure 4c shows optical microscope images of the fiber cross section. The fiber exhibits a good cylindrical shape and uniformity, with an outer diameter of 125 μm and inner diameter of 8 μm. The energy dispersive spectrum (EDS) measurements were employed to examine the cross section for element distribution. As depicted in Figure 4d, the elemental abundances of La and W show an obvious boundary of core-cladding, and the distribution circle of the Dy and Na ions are not so clear due to low element concentration, relatively. Meanwhile the Si and O elements are mainly distributed in the cladding region, which are consistent with the preform. The result indicates that the PF has a complete fiber structure, even if the elements have slightly undergone inter-diffusion at high temperature.

To achieve a high luminescent performance, we took a thermal approach by precisely controlling the thermal regime of nucleation and crystal growth to realize the in-suit preparation of the NaLa(WO_4_)_2_:Dy^3+^ nanocrystals in the core. Micro-Raman spectra were employed to investigate the microstructure of the fiber core region. As shown in the black curve in Figure 4e, it can be seen that there are no crystalline characteristic peaks occurring before heat-treatment, which implies that the PF core has an amorphous state. The CGFs are obtained after heat-treatment at a nucleation temperature of 700 °C for 2 h and a crystallization temperature at 940, 960, 980 and 1000 °C for 2 h (abbreviated as 700-2/940-2, 700-2/960-2 and so on), respectively. The bands around 340 and 915 cm^−1^ are observed in the Raman spectrum of the GCF cores, which are assigned to the υ_2_-υ_s_ (WO_4_) and υ_1_-υ_s_ (WO_4_) modes, respectively. When the heat-temperature is below 960 °C, the characteristic peaks tend to be sharper. After being heat-treated at 960 °C, the bands near 195, 331 and 787 cm^−1^ appear and are both attributed to the characteristic Raman bands of NaLa(WO_4_)_2_ [24]. Due to the thermal-induced interfacial bonding between nanocrystals and the glass matrix, a general decrease in the intensity of the characteristic band is observed at the processing temperatures from 980 °C to 1000 °C. The TEM image and selected area electron diffraction (SAED) pattern of the GCF (700-2/960-2) are shown in Figure 4f. It can be seen that the particles with a diameter of 4–6 nm are homogeneously dispersed in the glass. The HR-TEM image (Figure 4g) of a single nanoparticle exhibits the crystal lattice stripes with a space of 0.31 nm, according to the (112) direction of the NaLa(WO_4_)_2_ crystal.

**Figure 5:**
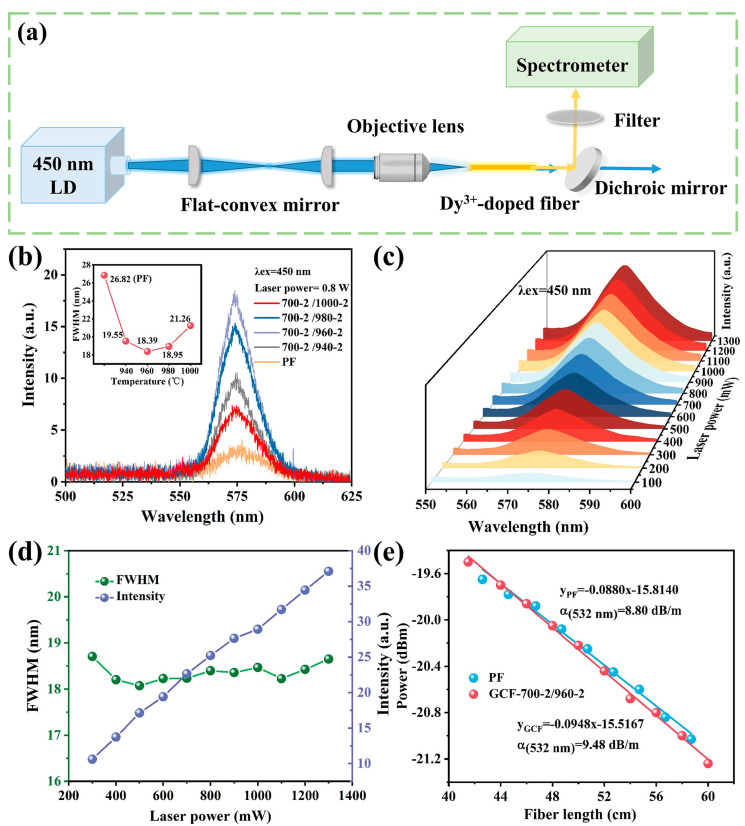
(**a**) Schematic of the yellow emission test; (**b**) emission spectra of the PF and GCFs at different temperatures of crystal growth (940–1000 °C) under 450 nm LD pumping; (**c**) yellow emission spectra of GCF(700-2/960-2); (**d**) its integrated intensity and FWHM under different pump power; and (**e**) optical transmission performance of the PF and GCF achieved at the heat-treatment temperature of 700 °C-2/960 °C-2 h.

As shown in the schematic of the yellow emission test in Figure 5a, the 450 nm laser beam was first shaped by two flax-convex mirrors and then coupled into the 10 cm long fiber through an objective lens. Finally, a dichroic mirror (HT@400~480 nm, HR@530~580 nm) was used to reflect the yellow signal light to the spectrometer for collection. Figure 5b presents the yellow emission spectra of the PFs and the GCFs under the excitation of a 450 nm LD laser. It can be observed that a more intense yellow emission is obtained from the GCFs than the PFs. In addition, it should be emphasized that the luminous intensity and FWHM (as depicted in Figure 5b insert) of the GCFs are closely related to the degree of crystallization. The GCF within the nanocrystals that were grown at 960 °C with the highest crystallinity (as shown in Figure 4e) exhibits the highest yellow light emission intensity, which is ≈5 times stronger than that of the PF. In addition, the reason for the change in FWHM may include two aspects. On the one hand, more Dy^3+^ enters the disordered crystal lattice and occupies different lattice sites, which is likely to cause an increase in bandwidth. On the other hand, the increase in crystallization degree with the rising heat-treatment temperature might cause the FWHM decrease. As displayed in Figure 5c,d, the subsequent experiment performed with the increase in the laser output power indicates that the integrated emission intensity of the GCF (700-2/960-2) became stronger, and at the same time, the FWHM remained relatively stable. The FWHM of that GCF is calculated to be ~18 nm in the yellow emission band and is overall larger than those in the reported traditional crystal and other disordered crystals (as listed in Table 3).

The propagation loss of the Dy^3+^-doped multi-material fibers was calculated using the following equation [32]:

(3) $$\alpha =-\frac{10\log\left(P_{out}/P_{in}\right)}{L}$$

where *P_out_* and *P_in_* are the output and input power, respectively, and *L* is the length of the fiber. Figure 5e depicts the cutback measurement of the PF and GCF (700-2/960-2). The propagation loss of the PF at 532 nm was calculated to be 8.80 dB/m. According to the study by Tick et al. [33], the scattering losses are minimal when nanocrystals have a size smaller than 1/20 of the light transmission wavelength. It can be seen that the NaLa(WO_4_)_2_:Dy^3+^ nanocrystals produce no noticeable transmission loss in the fiber. The above results further confirm that the Dy^3+^-doped NaLa(WO_4_)_2_ composite fiber is likely to realize a tunable yellow laser.

**Table 3:** Comparison of the FWHM of NaLa(WO_4_)_2_:Dy^3+^ GCFs with other Dy^3+^-doped crystals.

Materials	FWHM (nm) (at ~575 nm)	Ref.
Dy:Y_3_Al_5_O_12_	<7	[15]
Dy,Tb:LiluF_4_	<7	[16]
Dy:ZnWO_4_	6.50	[34]
Dy: Na_2_Gd_4_(MoO_4_)_7_	9.80(ϭ) 9.40(π)	[35]
Dy:CaYAlO_4_	~10	[36]
Dy: NaLa(WO_4_)_2_	13.72	this work
Dy: NaLa(WO_4_)_2_ GCFs	18.5~22	this work

## 4. Conclusions

In summary, Dy^3+^-doped NaLa(WO_4_)_2_ glass ceramic fiber with good uniformity was fabricated for the first time using the molten core method. The GCF displays efficient and broadband emissions in yellow spectra. Via the rational control of the thermal treatment, the emission band of the GCFs around 575 nm have a wide FWHM of 18~22 nm, which is remarkably higher than that of traditional crystals and disordered crystals reported. Meanwhile, the GCF heat-treated at 960 °C exhibits a five-times enhancement of luminescence intensity around 575 nm, compared with the PF. The present Dy^3+^-doped NaLa(WO_4_)_2_ GCF is a very promising material candidate for tunable yellow fiber lasers.

## Figures and Tables

**Table 1:** Refined crystallographic data for NaLa_0.95_(WO_4_)_2_: 0.05Dy^3+^.

Formula	*x* = 0	*x* = 0.05
Crystal system	Tetragonal	Tetragonal
Space group	I41/a	I41/a
a = b (Å)	5.359	5.355
c (Å)	11.656	11.649
α = β = γ (degree)	90	90
Volume (Å^3^)	334.606	334.102
Density (g/cm^3^)	6.572	6.548
R*_wp_* %	16.871	16.849
GOF	1.45	1.72

**Table 2:** NaLa_0.95_(WO_4_)_2_:0.05Dy^3+^ XRD Rietveld refinement of different atomic position.

Lable	Ion Type	Occupancy	x/a	y/b	z/c	Uiso * (Å^2^)
Na1	Na^+^	0.5	0.0	0.75	0.875	0.01
La1	La^3+^	0.45	0.0	0.75	0.875	0.01
W1	W^6+^	1.0	0.5	0.75	0.125	0.01
O1	O^2-^	1.0	0.736	0.60	0.044	0.01
Dy1	Dy^3+^	0.005	0.0	0.75	0.875	0.01

* Uiso: a parameter that characterizes the degree of atomic vibrations.

## Data Availability

The data presented in this study are openly available from the first author upon request.
